# Development and optimization of thermal contrast amplification lateral flow immunoassays for ultrasensitive HIV p24 protein detection

**DOI:** 10.1038/s41378-020-0168-9

**Published:** 2020-07-27

**Authors:** Li Zhan, Timothy Granade, Yilin Liu, Xierong Wei, Ae Youngpairoj, Vickie Sullivan, Jeff Johnson, John Bischof

**Affiliations:** 1 Department of Mechanical Engineering, University of Minnesota, Minneapolis, MN USA; 2 Centers for Disease Control and Prevention, Atlanta, GA USA; 3 Department of Biomedical Engineering, University of Minnesota, Minneapolis, MN USA

**Keywords:** Biosensors, Nanoparticles, Biosensors, Nanoparticles

## Abstract

Detection of human immunodeficiency virus (HIV) p24 protein at a single pg/ml concentration in point-of-care (POC) settings is important because it can facilitate acute HIV infection diagnosis with a detection sensitivity approaching that of laboratory-based assays. However, the limit of detection (LOD) of lateral flow immunoassays (LFAs), the most prominent POC diagnostic platform, falls short of that of laboratory protein detection methods such as enzyme-linked immunosorbent assay (ELISA). Here, we report the development and optimization of a thermal contrast amplification (TCA) LFA that will allow ultrasensitive detection of 8 pg/ml p24 protein spiked into human serum at POC, approaching the LOD of a laboratory test. To achieve this aim, we pursued several innovations as follows: (a) defining a new quantitative figure of merit for LFA design based on the specific to nonspecific binding ratio (BR); (b) using different sizes and shapes of gold nanoparticles (GNPs) in the systematic optimization of TCA LFA designs; and (c) exploring new laser wavelengths and power regimes for TCA LFA designs. First, we optimized the blocking buffer for the membrane and running buffer by quantitatively measuring the BR using a TCA reader. The TCA reader interprets the thermal signal (i.e., temperature) of GNPs within the membrane when irradiated by a laser at the plasmon resonance wavelength of the particle. This process results in higher detection and quantitation of GNPs than in traditional visual detection (i.e., color intensity). Further, we investigated the effect of laser power (30, 100, 200 mW), GNP size and shape (30 and 100 nm gold spheres, 150 nm gold-silica shells), and laser wavelength (532, 800 nm). Applying these innovations to a new TCA LFA design, we demonstrated that 100 nm spheres with a 100 mW 532 nm laser provided the best performance (i.e., LOD = 8 pg/ml). This LOD is significantly better than that of the current colorimetric LFA and is in the range of the laboratory-based p24 ELISA. In summary, this TCA LFA for p24 protein shows promise for detecting acute HIV infection in POC settings.

## Lateral flow immunoassays: HIV detection

A thermal contrast amplification variation of lateral flow immunoassays enables the ultrasensitive detection of the HIV p24 protein. The early detection of HIV relies on measuring p24 protein levels, as they appear in serum 7–10 days earlier than antibodies, another marker for HIV. Only small quantities of this protein are present, though. Lateral flow assays, however, suffer from poor detection limits, reducing their ability to sense the p24 protein. Here, researchers from University of Minnesota and Centers for Disease Control and Prevention enhance the detection limit of lateral flow immunoassays by using thermal contrast amplification to record the temperature change of membranes by irradiating gold nanoparticles with a laser. This thermal approach improved the limit of detection – reaching 8 pg/ml p24 protein – compared to the usual visual-based approach.

## Introduction

Globally, there were 1.7 million new human immunodeficiency virus (HIV) infection cases, leading to 37.9 million people infected in total by the end of 2018, predominantly in resource-limited areas (i.e., sub-Saharan Africa)^1^. Accurate diagnosis of HIV at point-of-care (POC) is the essential gateway to control the pandemic, especially in patients with acute infection before seroconversion occurs^2^. POC diagnostic technologies enable rapid screening of disease analytes in individuals outside of traditional laboratory settings. When combined with appropriate follow-up, POC testing can facilitate a faster diagnosis, leading to improved health outcomes^3,4^. Among the current commercial POC diagnostic devices, the lateral flow assay (LFA) is the most extensively used platform due to its speed, low cost, robustness, and ease of use^5,6^. LFA testing of HIV antibodies is widely employed in resource-limited areas and in nonclinical settings^7,8^. Nonetheless, HIV antibodies usually appear in blood ~21 days after infection. It is of considerable importance yet challenging to identify acute infections that carry high viral loads but are antibody negative at POC. In fact, in HIV-infected individuals, the p24 protein (i.e., viral capsid protein) is present in the serum or plasma 7–10 days earlier than the antibody^2,9^. The physiological concentration of p24 protein is in the pg/ml range and will first rise with time postinfection and then drop after antibody production is initiated^2^. Prompt diagnosis within this window period (i.e., within 21 days postinfection) can provide a critical opportunity to prevent transmission and facilitate effective antiretroviral treatments for potential cure^9^. In addition, successful early detection of p24 protein would be transformative in the diagnosis of infants whose mothers are HIV positive, where antibody tests of the infant are unreliable^10^.

**Fig. 1:**
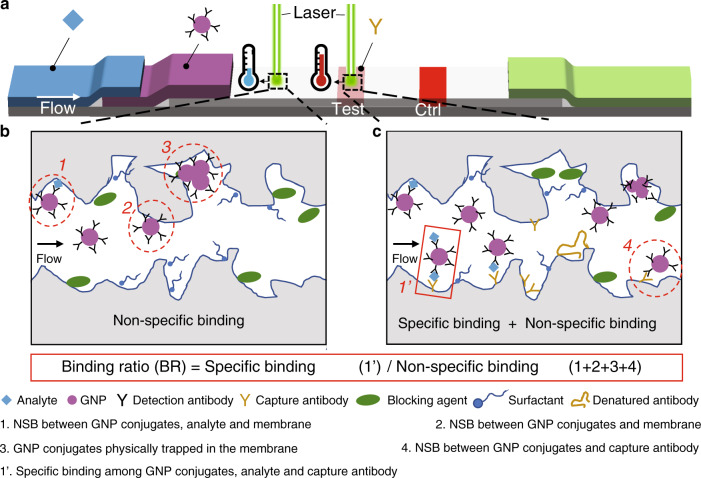
Thermal contrast amplification (TCA) reader can be used to probe specific binding (SB) and nonspecific binding (NSB) at various locations of LFA membrane. **a** Background and test line areas on LFA membrane are irradiated with a laser, resulting in different temperature increases due to distinct SB and NSB events and therefore establishing an SB/NSB ratio, which we defined as a new figure of merit called the binding ratio (BR). **b** For background area on the LFA membrane, analytes can nonspecifically bind to the nitrocellulose membrane, which could therefore capture GNP conjugates. NSBs occur between GNP conjugates with nitrocellulose membrane via hydrophobic and electrostatic interactions. In addition, aggregated GNPs can be trapped within the nitrocellulose pores. **c** For the test line area, SB refers to the sandwich interaction between the GNP conjugates, analyte and capture antibody on the membrane. Besides all the possible NSBs for the background area, another important NSB case in test line area is between GNP conjugates and the capture antibody

However, the poor limit of detection (LOD) for analytes of conventional LFAs (i.e., on the order of ng/ml) restricts their ability to detect p24 protein during acute infection (i.e., on the order of pg/ml); therefore, more complicated laboratory tests such as enzyme-linked immunoassay (ELISA) are required^11,12^. During traditional LFA tests (Fig. 1), the fluid sample is first added to the sample pad and then flows through the LFA by capillary forces, interacting with detection antibody-labeled gold nanoparticles (GNPs) from a conjugate pad and then capturing antibodies on a nitrocellulose membrane. Positive detection occurs if the target analytes in the sample are captured by antibody-GNP conjugates and anchored by the antibodies on the membrane, leading to accumulation of GNPs on the test line. The test line will develop color if enough GNPs are present. To significantly improve the LOD of traditional LFAs, various approaches have been developed to amplify the resultant signal and/or increase the specific binding (SB) of the sandwich structure involving the GNPs (Table S1). For instance, silver enhancement^13,14^, enzyme catalytic amplification^15,16^, surface-enhanced Raman scattering (SERS)^17,18^ and electrochemical amplification^19^ have been explored to provide 1–2 orders of magnitude LOD improvement by signal amplification. New particles such as larger GNPs^11^, magnetic particles^20,21^, and gold nanoshells^22^ have also been studied to increase SB and thereby enhance the detection signal. Furthermore, sample pretreatment methods including preconcentration assisted by dialysis^23^, magnetic field^24,25^ and electric field^12^ have also demonstrated the ability to boost SB at the test line. Numerous modifications to LFA design have been investigated to achieve LODs for p24 protein in the range of 0.8–50 pg/ml (Table 1), many of which still require multiple extra steps, trained personnel and complex equipment^16,26–28^. Therefore, a simple and rapid method leveraging the simplicity, low cost and robustness of existing LFAs with an ultrasensitive LOD (i.e., <10 pg/ml) would considerably benefit acute HIV diagnosis at POC^26,29^.

**Table 1:** p24 protein detection using laboratory and POC techniques

		LOD (pg/ml)	Detection range (pg/ml)	Detection methods	Assay Time (min)	Vendor/Reference
Laboratory	ELISA	4.69	4.69–300	Colorimetric	90	Abcam
		6.5	6.5–1500	Colorimetric	285	RayBiotech
		7.8	7.8–500	Colorimetric	270	R & D
		15.63	15.63–1000	Colorimetric	200	Sino
POC	CLIA-waived	25	N/A	Colorimetric	20–30	Abbott
	Literature	0.8	0.8–10000	Catalytic colorimetric	20	Ref. ^16^
		2	N/A	Fluorescent	40	Ref. ^27^
		30	30–1000	Magnetic	40	Ref. ^28^
		50	50–1000	Colorimetric	40	Ref. ^26^

Importantly, despite all the methods for enhanced LFAs, nonspecific binding (NSB) is one of the major limiting factors for detecting lower analyte concentrations^9^. The LOD is defined as the lowest analyte concentration to be distinguished from the negative samples containing no analyte at a certain confidence level (i.e., >95%)^30^. The signals from negative samples can be attributed to the NSB, which can deteriorate the LOD of the assay. For example, as the final signal is contributed by both SB and NSB, at low analyte concentrations, if NSB dominates the competition with SB, the resultant signal will not be distinguishable from the negative samples. This issue can be addressed by reducing NSB and/or improving the resolution of signal detection methods to differentiate the SB signal. For GNP-based LFAs, various NSBs occur on the nitrocellulose membrane (Fig. 1). In areas outside the test and control lines, NSB between antibody-GNP conjugates and the nitrocellulose membrane is the major cause, which includes but is not limited to the hydrophobic antibody-nitrocellulose membrane interaction and electrostatic interaction between the exposed GNP surface and nitrocellulose membrane. Inside the test line area, additional NSB between antibody-GNP conjugates and the capture antibody leads to higher signals for negative samples. In addition, GNP conjugates and possibly GNP aggregates can be physically trapped inside the nitrocellulose membrane during the flow. Furthermore, analytes can nonspecifically bind to the nitrocellulose membrane, which could therefore capture GNP conjugates. To reduce these NSBs, blocking of the nitrocellulose membrane, surface blocking of GNP conjugates and optimization of the running buffer need to be performed. Specifically, nitrocellulose membrane blocking involves preparing the membrane in buffers containing surfactant and inert macromolecules. Bovine serum albumin (BSA) is the most popular inert macromolecule to block free GNP conjugate surfaces. The pH of the running buffer and inclusion of surfactant and macromolecules (i.e., protein and/or polymer) need to be optimized. Importantly, some treatments to reduce NSB can also impair the SB between the analyte and antibodies. These optimization procedures are normally conducted in a trial-and-error manner. For instance, after testing various membrane blocking buffers, researchers choose those with “clean” backgrounds and strong test and control lines based on the visual color intensity for further iterations. However, this approach fails to provide definitive conclusions and rule out cases with subvisual signals, leading to excessive experimentation and suboptimal final performance.

In this report, we present the development and thorough optimization of a thermal contrast amplification (TCA) LFA for ultrasensitive p24 protein detection. The TCA reader measured the temperature changes of the membrane with GNPs when irradiated by a laser using an infrared camera. The temperature changes are proportional to the GNP number concentration (i.e., # GNPs/mm^3^). In addition, as infrared cameras can accurately detect slight changes in temperature (i.e., 0.1 °C), subvisual GNPs within the membrane can be quantitatively detected, leading to enhanced thermal LOD compared with visual LOD. To develop ultrasensitive TCA LFAs, we pursued several innovations as follows: (a) defining a new quantitative figure of merit for LFA design based on a specific to nonspecific binding ratio (BR) of GNPs; (b) using different sizes and shapes of GNPs in systematic optimization of TCA LFA designs; and (c) exploring new laser wavelength and power regimes for TCA LFA designs. First, we demonstrated a simple and novel strategy to improve the LOD by optimizing BR as the figure of merit during screening of the membrane blocking buffer and assay running buffer. Based on the BR, we rapidly identified the optimal buffer recipes and confirmed a fourfold improvement in the thermal LOD using recipes with optimal BR compared with recipes selected using traditional methods such as the naked eye. Furthermore, to maximize the performance of TCA LFAs, we studied the effect of laser power, laser wavelength, and GNP size and shape on the LOD. We showed that 8 pg/ml p24 protein spiked in human serum can be detected with TCA LFAs using 100 nm gold spheres and a 100 mW 532 nm laser. Overall, we developed an ultrasensitive TCA LFA by optimizing the designs of BRs, GNPs and lasers. This TCA LFA with ultrasensitive p24 protein detection holds great promise in improving acute HIV infection diagnosis at POC.

## Results and discussion

### GNP synthesis

**Fig. 2:**
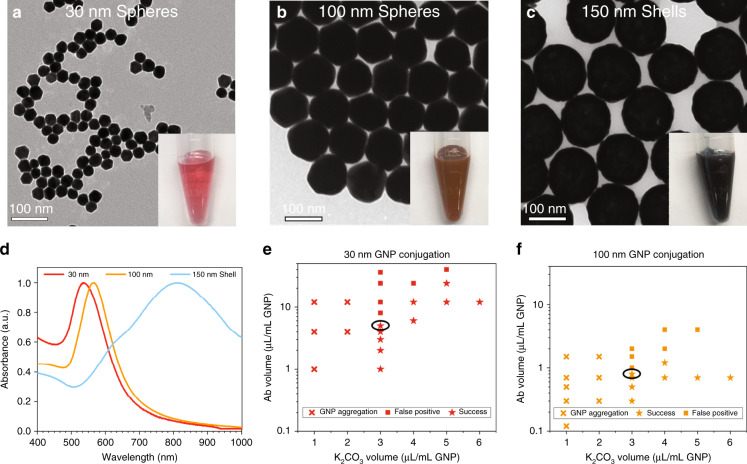
Characterization of various GNPs and optimization of antibody conjugation. **a**–**c** TEM images of 30 and 100 nm gold nanospheres and 150 nm gold nanoshells. The insets are the images of GNP solution showing red, muddy brown and blue colors for 30 and 100 nm gold nanospheres and 150 nm gold nanoshells, respectively. **d** Normalized UV-Vis-NIR spectrum of 30 nm and 100 nm gold nanospheres and 150 nm gold nanoshells. **e**, **f** Optimization of GNPs (30 and 100 nm)—1E5 antibody passive absorption by varying the amount of 0.2 M K_2_CO_3_ and antibody added to 1 ml GNP as-synthesized solution. Success conditions (star symbols) avoid GNP aggregation (cross symbols) and severe nonspecific bindings at test line (i.e., false positives, square symbols). The circled success conditions provide strongest control line (i.e, specific binding) with minimal consumption of antibody

Citrate-stabilized 30 and 100 nm GNP spheres with highly uniform size distributions were synthesized using the seed-mediated growth method. Analysis from TEM images revealed the GNP size to be 28.6 ± 0.8 nm and 98.2 ± 2.4 nm for the 30 and 100 nm spheres, respectively (Fig. 2a, b). The size of nanoshells purchased from nanoComposix is 146 ± 4.7 nm (Fig. 2c). The normalized spectra of the three GNPs are shown in Fig. 2d. The resonant oscillation of free electrons in gold with incoming light, known as surface plasmon resonance (SPR), greatly enhances the optical properties (scattering and absorption) of GNPs^31^. The UV–Vis spectrometer measures the extinction (i.e., sum of scattering and absorption) of GNP solutions. The peak extinction (shown in Fig. 1d as absorbance) wavelengths are 534 nm, 565 nm and 810 nm for 30 nm spheres, 100 nm spheres and 150 nm shells, respectively. The unique optical properties render different colors for various GNPs. For instance, aqueous solutions of 30 nm spheres, 100 nm spheres and 150 nm shells are pink-red, muddy brown and blue (Fig. 2). To maximally harness the GNP photothermal conversion ability (i.e., absorption), lasers with a wavelength matching with GNP spectrum peaks should be used. For example, we chose a 532 nm laser for 30 nm and 100 nm spheres and an 800 nm laser for 150 nm shells.

### GNP – 1E5 mAb conjugations

The GNP conjugate plays a vital role in determining the ultimate LOD and specificity of LFAs. Successful GNP conjugates should have specific and high binding affinity to the target analyte to achieve a low LOD and low nonspecific interactions with the nitrocellulose membrane and test line antibodies to avoid false positives (i.e., high specificity). For citrate-stabilized GNPs, the antibody can adsorb strongly to the GNP surface to form stable conjugates while retaining its binding activity. In general, this passive conjugation can be attributed to electrostatic interactions (i.e., negatively charged GNP surface and positively charged sites on the antibody), hydrophobic attraction (i.e., the antibody and the gold surface) and covalent bonding between the gold atoms and sulfur atoms from the antibody^32^. Electrostatic and hydrophobic interactions are usually the major considerations for optimal conjugation. To achieve this aim, the pH needs to be maintained at or slightly higher than the isoelectric point of the antibody. The isoelectric point is the pH at which a molecule carries no net electrical charge. At higher pH values, the antibody carries a net negative charge. Positively charged antibodies will lead to aggregation among negatively charged GNPs. Neutral or slightly negatively charged antibodies facilitate binding to the GNP surface and maintain interparticle stability. The pH can be tuned by adding 0.2 M K_2_CO_3_ to the GNP solution before introducing the antibody. Another important parameter is the amount of antibody used. A higher surface antibody coating density provides a stronger SB. However, if overcoated with antibodies, GNP conjugates can increase NSB at the test line and therefore cause false positives. To reduce this NSB, BSA is commonly added to block the free surfaces on GNPs.

We optimized the 1E5 antibody-GNP (30 and 100 nm) conjugation by varying the pH and the added 1E5 volume. For 1 ml as-synthesized GNPs, 1–6 μl 0.2 M K_2_CO_3_ was added to adjust the pH from 5 to 9. The 1E5 volume ranging from 0.1 to 10 μl was added. As shown in Fig. 2e, f, the results achieved by varying antibody vs. K_2_CO_3_ addition range from failure due to GNP aggregation and false positives to eventual success. For instance, at a low K_2_CO_3_ volume (i.e., pH < 7), GNPs aggregated, likely due to the positive charge of antibodies below the isoelectric point. At a medium K_2_CO_3_ volume (i.e., pH ~ 7), GNP conjugates were stable and provided a clear red color on the control lines consisting of p24 protein, indicating successful conjugations. Notably, the control line color intensity increased with increasing antibody volume as a result of stronger SB. However, when the antibody volume for conjugation was too high, false positives were noted (i.e., failure), possibly due to increasing NSB between GNP conjugates and test line antibodies. Finally, at a high K_2_CO_3_ volume (i.e., pH > 7), a higher antibody volume was required to obtain an equivalent control line as when the pH was ~7, suggesting suboptimal conjugation and unneeded antibody. For the following LFA optimization, we therefore used conjugation conditions for 3 μl 0.2 M K_2_CO_3_ adjusted to pH = ~7 and the maximal allowable volume of antibody prior to showing false positives.

### Nitrocellulose membrane blocking buffer optimization

The membrane is the critical component of LFAs, and nitrocellulose membranes are most commonly used due to their adjustable pore size, high protein binding capacity and controllable manufacturing procedures^33^. Typically, proprietary chemical coatings from the manufacturer were added to modify the hydrophobic nitrocellulose to be hydrophilic for capillary flow. Test line and control line proteins bind to the nitrocellulose membrane by electrostatic and hydrophobic interactions. In addition, GNP conjugates and even analytes can nonspecifically bind to the nitrocellulose membrane due to their protein-binding properties, causing a stained background. To block these extra protein binding sites, the membrane is usually soaked in blocking buffer consisting of macromolecules and surfactants before LFA assembly. On the other hand, blocking agents can also be added to the running buffer to block the membrane during LFA tests. A high concentration of blocking agents can reduce the NSBs but also interfere with the SB, therefore affecting the LFA sensitivity and performance (SB/NSB ratio). It is important to quantitatively compare the effects of various blocking solutions on the SB/NSB ratio in LFAs. This quantitative comparison is impossible by tracking the color intensity of GNPs on the membrane background, as it can be subvisual, and the difference is below the resolution of a scanner or the naked eye.

**Fig. 3:**
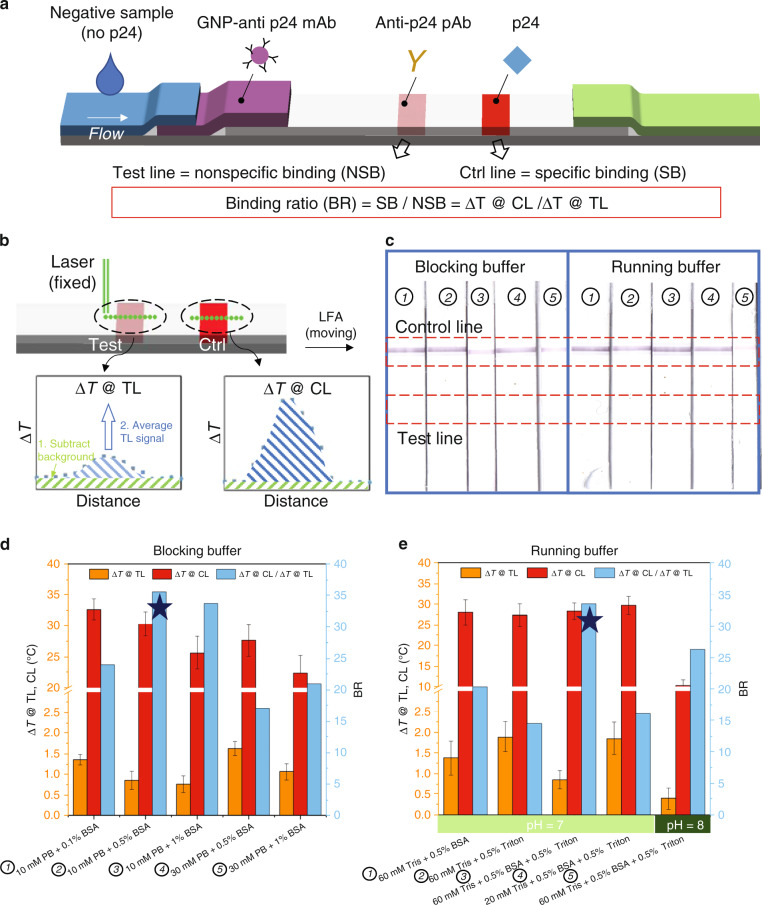
Quantitative optimization of membrane blocking buffer and running buffer for TCA LFA. **a** The architecture of TCA LFA used for SB/NSB optimization. **b** TCA reader will scan the test line and control line areas separately. The resultant temperature curve was analyzed in two steps: the background temperature change was first averaged and then subtracted from the average test line (or control line) temperature change. **c** Images of LFA tested with various membrane blocking buffers and running buffers using negative p24 samples. Note that the test line and background stain were all invisible (i.e., subvisual). **d** Test line temperature signal (Δ*T* @ TL), control line temperature signal (Δ*T* @ CL) and their ratio (i.e., SB/NSB ratio) were plotted against various membrane blocking buffers. Δ*T* @ TL represents NSB as negative p24 sample was used and Δ*T* @ CL represents SB as p24 was sprayed as the control line. **e** Δ*T* @ TL, Δ*T* @ CL and their ratio were plotted against various running buffers. The buffer with highest SB/NSB ratio was marked with a star

As SB and NSB always coexist on test lines for positive p24 samples, by using negative samples and spraying p24 on the control line, we can decouple the SB (i.e., control line) and NSB (i.e., test line) in individual LFA tests. Therefore, the ratio of the control line signal to the test line signal represents the SB/NSB ratio (Fig. 3a). In Fig. 3b, we used the TCA reader to quantitatively obtain GNP distribution across the test and control lines after testing the LFAs with running buffer precluding p24 protein. To obtain sufficient temperature change (i.e., Δ*T*) while avoiding burning the membrane, laser powers of 30 and 10 mW were used for the test line and control line scans, respectively. To interpret the resultant temperature change curves shown in Fig. 3b, we first divided the curve into two parts: background area and test (or control) line area. NSB categories 1 (between GNP conjugates and membrane) and 2 (GNP conjugates physically trapped in the membrane) indicated in Fig. 1b can be extrapolated from the temperature rise in the background area. Similarly, the extra temperature increases in the test line represent NSB category 3 (between GNP conjugates and test line antibody), as indicated in Fig. 1b. To obtain the final thermal signal at the test line, we first average the Δ*T* of the background area and then subtract it from the average Δ*T* of the test line area. Therefore, this test line signal will be used to represent NSB category 3 when a negative sample is tested. A similar process was performed to the control line temperature curve to obtain the SB.

We used 30 nm sphere conjugates for optimization of the membrane blocking buffer and running buffer. To reduce interference with SB, we chose to use minimal blocking agents. We added various concentrations (i.e., 0.1, 0.5, and 1%) of BSA to phosphate buffer (PB) at various ionic concentrations (i.e., 10 and 30 mM). A running buffer composed of 60 mM Tris, 0.5% BSA and 0.5% Triton (pH = 7) was used. Images of LFA strips are shown in Fig. 3c. It is important to note that most strips appear similar based on visual intensity (i.e., naked eye) inspection. Fig. 3d demonstrates the advantages of TCA to quantitatively compare SB and NSB when using different membrane blocking buffers. For instance, for 10 mM PB, the addition of 0.1% BSA led to higher NSB than with 0.5 and 1% BSA; however, 1% BSA led to a decrease in SB. Thus, the addition of 0.5% BSA provides the highest BR when 10 mM PB is used. In addition, with the same BSA concentration, increasing the PB concentration from 10 to 30 mM reduces the BR by both increasing NSB and decreasing SB. There have been studies reporting that increasing ionic strength tends to reduce antibody-antigen binding constants^34^. The increasing NSB at high ionic strength may be caused by the changes in GNP conjugates and requires further investigation. Collectively, we identified 10 mM PB + 0.5% BSA with the highest BR as our membrane blocking buffer for further optimization steps.

### Running buffer optimization

The running buffer is another critical component to modulate and reduce NSB. Various salts, surfactants and macromolecules are usually included in the running buffer. In general, the running buffer needs to be optimized for each individual LFA. For the buffer components, we tested various combinations of Tris (20 and 60 mM), BSA (0 and 0.5%) and Triton X-100 (0 and 0.5%) at different pH values (7 and 8). GNP conjugates (30 nm) and membranes blocked with 10 mM PB + 0.5% BSA were used. Again, simple visual inspection based on color intensity fails to compare various running buffers quantitatively (Fig. 3c). As indicated in Fig. 3e, the combination of BSA and Triton X-100 resulted in lower NSB (i.e., Δ*T* @ TL) than that with BSA or Triton X-100 alone. Increasing the pH from 7 to 8 greatly reduced the NSB while also impairing the SB. Furthermore, reducing the Tris concentration from 60 to 20 mM caused an increase in NSB. Therefore, 60 mM Tris + 0.5% BSA + 0.5% Triton (pH = 7) outperformed the other buffers, exhibiting the highest BR (i.e., Δ*T* @ CL/Δ*T* @ TL). Indeed, these trends may be antibody dependent and may vary from assay to assay, as each individual LFA uses different antibodies. Each monoclonal antibody or set of polyclonal antibodies may behave differently in response to changes in the buffer composition, GNP conjugation conditions, nitrocellulose membrane, etc. Importantly, the TCA reader allows quantitative analysis of GNP distribution within individual LFAs, which can provide valuable information on how those SBs and NSBs change under different assay configurations.

### Optimizing LOD of p24 TCA LFA

To maximize the performance of TCA LFAs, we studied important parameters including laser power, GNP size and shape, and laser wavelength. Regarding various GNP sizes and shapes, we found in our previous work that two orders of magnitude LOD enhancement in LFA can be achieved by replacing visual color intensity detection of traditional 30 nm spheres with TCA reader detection of 100 nm spheres^11^. It was later confirmed by other studies that larger GNPs can indeed improve the detection limit in LFAs^16,35,36^. In addition, we reported that further increasing the gold nanosphere size above 100 nm could lead to settling due to the heavy mass of the gold nanospheres, therefore diminishing the benefits of larger sizes beyond 100 nm^11^. Gold nanoshells with silica cores can reduce the nanoparticle weight while maintaining a large size for better optical properties and a higher antibody load for superior binding. This improvement is due to the lighter density of silica (i.e., 2.65 g/cm^3^) than gold (19.32 g/cm^3^), where a 10 nm thick gold shell layer can be tuned into the NIR range (i.e., 780–2500 nm). This approach will reduce the laser absorption from the nitrocellulose membrane and LFA backing when an NIR laser is used for the TCA reader.

**Fig. 4:**
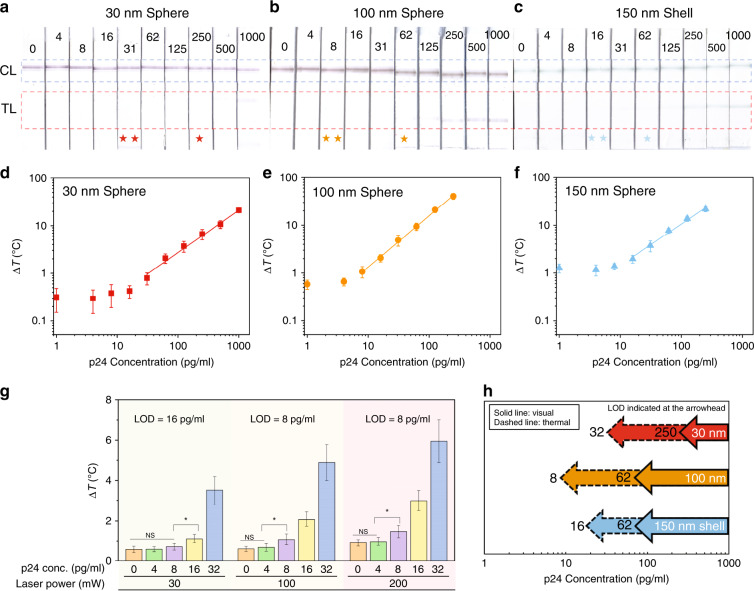
Ultrasensitive p24 detection with an optimized LFA and TCA reader. **a**–**c** Images of p24 LFAs tested with twofold serial dilutions of p24 using 30 nm spheres, 100 nm spheres and 150 nm shells, with visual LODs of 250, 62 and 62 pg/ml, respectively. The numbers indicated in the image are the p24 concentrations in pg/ml. Visual and thermal LODs are marked with one star and two stars, respectively. **d**–**f** Quantitation of p24 concentration in LFAs with the TCA reader. The thermal LODs for LFAs with 30 nm spheres, 100 nm spheres and 150 nm shells are 32, 8 and 16 pg/ml, respectively. **g** For LFAs with 100 nm spheres, increasing the TCA reader laser power from 30 to 100 mW improved the LOD from 16 to 8 pg/ml. Further increasing the laser power to 200 mW resulted in the same LOD as that with 100 mW laser power, revealing that NSB dominates SB at low p24 concentrations (i.e., <8 pg/ml) and that increasing the laser power fails to detect the SB. *p* < 0.05. **h** Summary of visual and thermal LODs of p24 LFAs with 30 nm spheres, 100 nm spheres and 150 nm shells

Here, we selected 150 nm gold nanoshells with silica cores together with 30 and 100 nm gold nanospheres for comparison of the LOD in p24 TCA LFAs. The optimal nitrocellulose membrane blocking buffer and running buffer formulations are indicated in Fig. 3. Negative samples (i.e., running buffer without p24) were first tested to establish the thermal signal of negative controls. LFAs with 30 nm spheres, 100 nm spheres and 150 nm shells were then tested with a twofold serial dilution of p24 first using running buffer. The images of these LFAs with labeled p24 concentrations are shown in Fig. 4a–c. The visual detection limits are 250, 62, and 62 pg/ml for 30 nm spheres, 100 nm spheres and 150 nm shells, respectively.

Next, we explored whether increasing the laser power can further improve the LOD because the heat generation by GNPs is proportional to the laser power^24^. Different laser powers, namely, 30, 100 and 200 mW, were used to irradiate 100 nm sphere LFAs with various p24 concentrations from 0 to 32 pg/ml. To determine the LOD, we chose *k* = 3 in Eq. 1, meaning that the LOD sample will be above the average thermal signal of the negative sample plus three times its standard deviation. As the laser power increased from 30 to 100 mW, the LOD decreased from 16 to 8 pg/ml, yet remained at 8 pg/ml when the laser power was doubled to 200 mW (Fig. 4g). This result indicates that increasing the laser power can improve the LOD to a certain extent, but the limiting factor for an improved p24 LOD is the lack of specifically bound GNPs at the test line that can be distinguished from nonspecifically bound GNPs (i.e., negative samples) using TCA. Additionally, we investigated the effect of laser wavelength on the enhanced performance of TCA LFAs. Specifically, with the laser power set to 100 mW, 532 nm and 800 nm lasers were used separately for TCA LFAs based on 30 nm spheres, 100 nm spheres and 150 nm shells. Indeed, the LOD enhancement (i.e., compared with visual detection) reached a maximum when the laser wavelength matched the GNP spectrum peak, for example, the 532 nm laser for 30 nm and 100 nm spheres and the 800 nm laser for 150 nm shells (Table S2). Moreover, we performed p24 testing with 30 nm spheres using suboptimal buffer selections suggested by traditional methods (i.e., the naked eye). For example, based on the color intensity of the test and control lines shown in Fig. 3b, membrane blocking buffer 1 (i.e., 10 mM PB + 0.1% BSA) and running buffer 1 (60 mM Tris +0.5% BSA) showed strong control lines and negligible test lines. In this case, we demonstrated that a 4-fold lower thermal LOD can be achieved with optimal buffer selection (i.e., membrane blocking buffer 2 and running buffer 3) using BR as the figure of merit (Fig. S1). This result demonstrated that our new strategy of BR optimization can simplify the LFA development process and provide better ultimate LFA performance (Table S1).

For the comparison of various GNPs, the laser power was set to 100 mW. The results are plotted in Fig. 4d–f for three different GNPs. The thermal LODs are 32, 8, and 16 pg/ml for 30 nm spheres, 100 nm spheres and 150 nm shells, respectively (Fig. 4h). In particular, the TCA reader demonstrated quantitation of p24 with all GNP types. If one were able to continue to increase the GNP size without settling, one may expect the LOD improvement to continue. Nevertheless, this did not occur with the 150 nm shells, as a lower LOD occurred with the 100 nm spheres instead. Specifically, a higher temperature change (i.e., 1 °C) at the test line (after subtracting the background) in the 150 nm-shell LFA was noticed compared with the 100 nm-sphere LFA (i.e., 0.5 °C), indicating increased NSB between nanoshell conjugates and test line antibodies (Fig. 4e, f). In all cases, we showed that the SB/NSB ratio plays a determining role in detecting the lowest possible analyte concentration using TCA LFAs. To demonstrate the practical usage of our ultrasensitive p24 TCA LFA, we spiked p24 protein into human serum and performed a dilution test using 100 nm sphere TCA LFAs and a 100 mW 532 nm laser. We confirmed that the thermal LOD remained unchanged compared with spiked buffer at 8 pg/ml, indicating that our TCA LFAs performed well in a complex biological environment (Fig. S2).

## Materials and methods

### Materials and reagents

For the synthesis of 30 and 100 nm gold nanospheres, sodium citrate tribasic dihydrate, hydrogen tetrachloroaurate (HAuCl_4_), and hydroquinone were purchased from Sigma (St. Louis, MO). Carboxyl gold nanoshells (150 nm) with a silica core were purchased from nanoComposix (San Diego, CA). For LFA fabrication, a CN 95 nitrocellulose membrane was obtained from Sartorius (Bohemia, NY). The sample pad, conjugate pad and wicking pad were purchased from GE Healthcare (Chicago, IL). A mouse monoclonal antibody (mAb) against p24 (referred to as 1E5) was developed by the CDC^36^. Polyclonal rabbit anti-p24 antibody was commercially produced (Bio-Synthesis Inc, Lweisville, TX) and purified before use by affinity chromatography. Recombinant p24 was purchased from Fitzgerald Industries International (Acton, MA).

### GNP synthesis and conjugation

To synthesize monodispersed 30- and 100-nm GNPs, seed-mediated growth methods were used^37^. Briefly, 15 nm GNP seeds were first synthesized by adding 1 ml 3% (w/v) sodium citrate to 100 ml boiling 0.25 mM HAuCl_4_ under vigorous stirring. The 30 and 100 nm GNPs were then synthesized by mixing the sodium citrate, HAuCl_4_, and 15 nm seeds followed by addition of hydroquinone for the reduction of ionic gold. After synthesis, the GNPs were characterized by UV–visible spectroscopy (Synergy HT, BioTek) and transmission electron microscopy (TEM, Tecnai G2).

Passive absorption of 1E5 mAb to 30 and 100 nm GNPs was performed. Specifically, to obtain the optimal conjugation, various volumes of 0.2 M K_2_CO_3_ were added to 1 ml as-synthesized GNP solutions. Then, various amounts of 1E5 mAb were added to the mixture. The solution was vortexed and incubated at 4 °C for 2 h. The free GNP surfaces were blocked with 1% BSA for 0.5 h at 4 °C. The mixture was washed through centrifugation for 20 min at 3000 and 400 × *g* for the 30 and 100 nm GNPs, respectively, to remove the unbound proteins and then was suspended in 10 mM phosphate buffer with 0.5% BSA and 3% sucrose. 1E5 antibodies were covalently linked to the 150 nm GNS through EDC/Sulfo-NHS chemistry following protocols provided by nanoComposix.

### TCA LFA fabrication and optimization

Polyclonal rabbit anti-p24 antibodies (2 mg/ml) and recombinant p24 (20 μg/ml) were stripped onto nitrocellulose membranes as test lines and control lines, respectively. The membrane was then blocked with different recipes of blocking buffer consisting of phosphate buffer and BSA. The conjugate pad, membrane and absorbent pad were assembled onto a polyester adhesive backing, allowing 1 ~ 2 mm overlap between adjacent components. The assembly was cut into 3 mm width individual strips using a Kinematic 2360 programmable shearer (Kinematic Automation, Twain Harte, CA). Then, 10 μL 1E5 conjugated GNPs were applied onto the conjugate pad. Different running buffers composed of Tris, BSA and Triton at various concentrations and pH values were tested for the optimal SB/NSB ratio (Fig. 3).

### Thermal contrast reading

A TCA reader reported previously was used to collect the temperature signal of the developed LFAs to guide the assay optimization efforts^38^. First, the tested LFA was placed on a 3D-printed holder and inserted into the TCA reader. Then, the reading area, for example, the location of the area of interest, was typed in the software. With the positions of the laser and IR camera fixed, the stepper motor moved the holder (i.e., the LFA) forward for the next reading after the temperature signal at the previous location was captured by the IR camera. To collect the thermal signals of the test line, we set the TCA measurement length to 2.5 mm, including the test line (~1 mm) and adjacent background area (~1.5 mm), across the centerline of the LFA. The distance between reading spots is 0.125 mm (Fig. 3a). Thermal signals of control lines were obtained in the same manner. The laser power can be adjusted from 10 to 500 mW. Finally, the temperature rise at different locations on the membrane was plotted. The thermal signals at the test line and/or control line were analyzed in a two-step manner: the background temperature change was first averaged and then subtracted from the average test line (or control line) temperature change (Fig. 3a). To investigate the impact of the laser wavelength used in the TCA reader for different GNP types, we compared the LOD enhancement (i.e., thermal vs visual) when different laser wavelengths (532 or 800 nm) were used for various GNPs (30 nm gold spheres, 100 nm gold spheres, or 150 nm gold-silica shells).

### p24 dilution test and LOD determination

A 100 µL twofold serial dilution of p24 spiked into running buffer was tested with optimized LFA designs with 30 nm and 100 nm spheres and 150 nm shells. To define the detection limits for thermal contrast reading, we tested three negative samples to establish the baseline and performed three replicates for each positive sample. The detection limit of the thermal analysis was defined according to the IUPAC method as the lowest concentration tested whose thermal signal (Δ*T*) satisfies the following relation:

1 $$\Delta T\, > \,\Delta T_B + k \cdot s_B$$

where Δ*T*_*B*_ is the mean of the negative sample signal, *s*_*B*_ is the standard deviation of the negative sample signal, and *k* is the numerical factor in accordance with the confidence level (i.e., *k* = 3 for 95% confidence interval)^39,40^.

In addition, p24 protein was spiked into human serum and tested with TCA LFA using 100 nm gold spheres and a 100 mW 532 nm laser.

## Supplementary information

supplemental information for MICRONANO-01135R
