# Supplementary material for: Development and optimization of thermal contrast amplification lateral flow immunoassays for ultrasensitive HIV p24 protein detection
Source: Microsyst Nanoeng. 2020 Jul 27;6:54. doi: 10.1038/s41378-020-0168-9 (PMC8433161; doi:10.1038/s41378-020-0168-9)
Supplement: Supplementary file 1 — supplemental information for MICRONANO-01135R [file 41378_2020_168_MOESM1_ESM.docx]

**Development and optimization of thermal contrast amplification lateral flow immunoassays for ultrasensitive HIV p24 protein detection**

**Supplemental Information**

Li Zhan,^1^ Timothy Granade,^2^ Yilin Liu,^1^ Xierong Wei,^2^ Ae Youngpairoj,^2^ Vickie Sullivan,^2^ Jeff Johnson,^2^ John Bischof^1,3^

^1^ Department of Mechanical Engineering, University of Minnesota, Minneapolis, Minnesota, USA

^2^ Centers for Disease Control and Prevention, Atlanta, Georgia, USA

^3^ Department of Biomedical Engineering, University of Minnesota, Minneapolis, Minnesota, USA

**Table S1. Methods to improve LOD of LFAs**

| **Strategies** | **Detection label** | **Extra steps?** | **Optimized BR (SB/NSB) ^a^** | **Ref** |
| --- | --- | --- | --- | --- |
| Silver enhancement | GNP ^b^ | Yes, silver staining | no | 1,2 |
| SERS ^c^ | GNP | Yes, Raman signal collection | no | 3,4 |
| Enzyme catalytic amplification | GNP-HRP ^d^ | Yes, enzymatic amplification | no | 5 |
| Fluorescent enhancement | Fluorescent dye | Yes, fluorescent imaging | no | 6 |
| Platinum nanocatalyst amplification | PtNC ^e^ | Yes, oxidation of CN/DAB substrate ^f^ | no | 7 |
| Quantum dot enhancement | Quantum dot | Yes, UV-photometer imaging | no | 8 |
| Magnetic signal enhancement | Magnetic particles | Yes, magnetic signal reader | no | 9 |
| SB/NSB ratio optimization and TCA ^g^ | Various GNP size and shape | Yes, TCA reader | Yes | This work |

a. SB: specific binding; NSB: non-specific binding

b. GNP: gold nanoparticle

c. SERS: surface-enhanced Raman scattering

d. HRP: horseradish peroxidase

e. PtNC: porous platinum core-shell nanocatalysts

f. CN/DAB: (4-chloro-1-naphthol/3,3′-diaminobenzidine, tetrahydrochloride)

g. TCA: thermal contrast amplification

**Table S2. LOD enhancement using TCA reader with different laser wavelength for various GNP size and shape ***

|  | **532 nm laser **** | **800 nm laser** |
| --- | --- | --- |
| 30 nm gold spheres | 8-fold | 2-fold |
| 100 nm gold spheres | 8-fold | 2-fold |
| 150 nm gold-silica shell | 2-fold | 4-fold |

* the LOD enhancement was compared to visual detection, a 2-fold serial dilution of p24 was performed.

** the power of both 532 nm and 800 nm laser was set to 100 mW for this comparison.


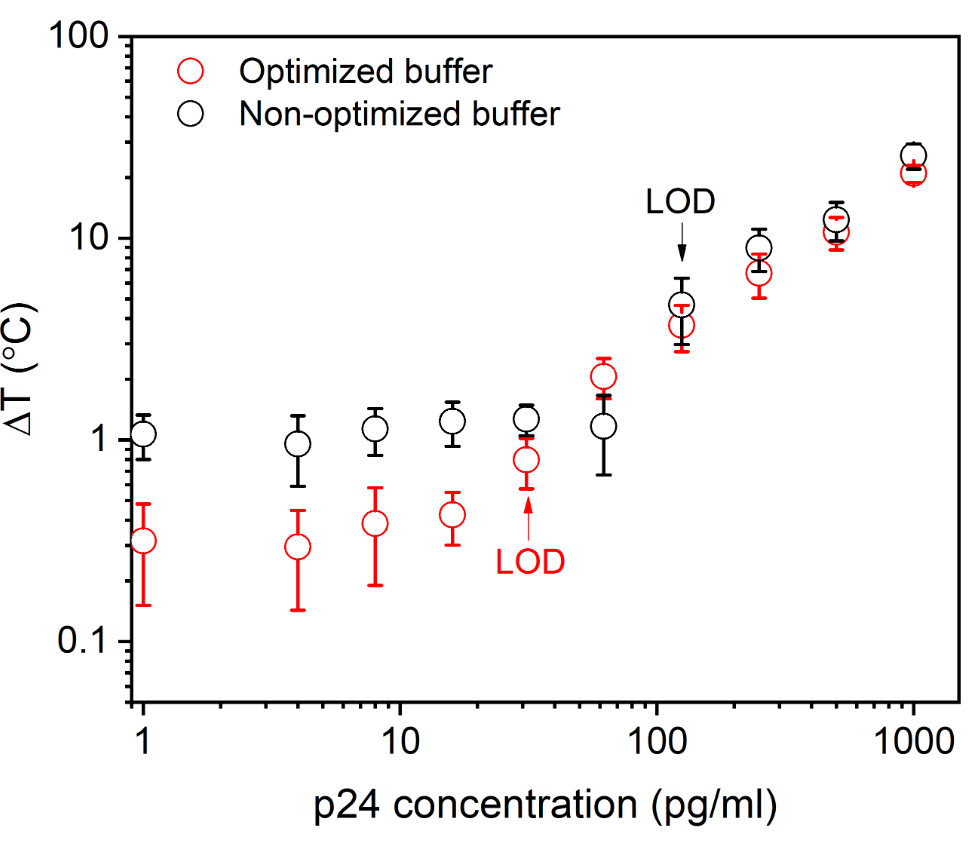


**Figure S1**. 4-fold improvement in thermal LOD can be achieved by using recipes with optimized BR (i.e., membrane blocking buffer 2 and running buffer 3 in Figure 3) compared to recipes (i.e., membrane blocking buffer 1 and running buffer 3 in Figure 3) selected using traditional methods such as naked eyes. LODs were indicated by the arrows. In these tests 30 nm spheres were used in TCA LFAs irradiated by 100 mW 532 nm laser.


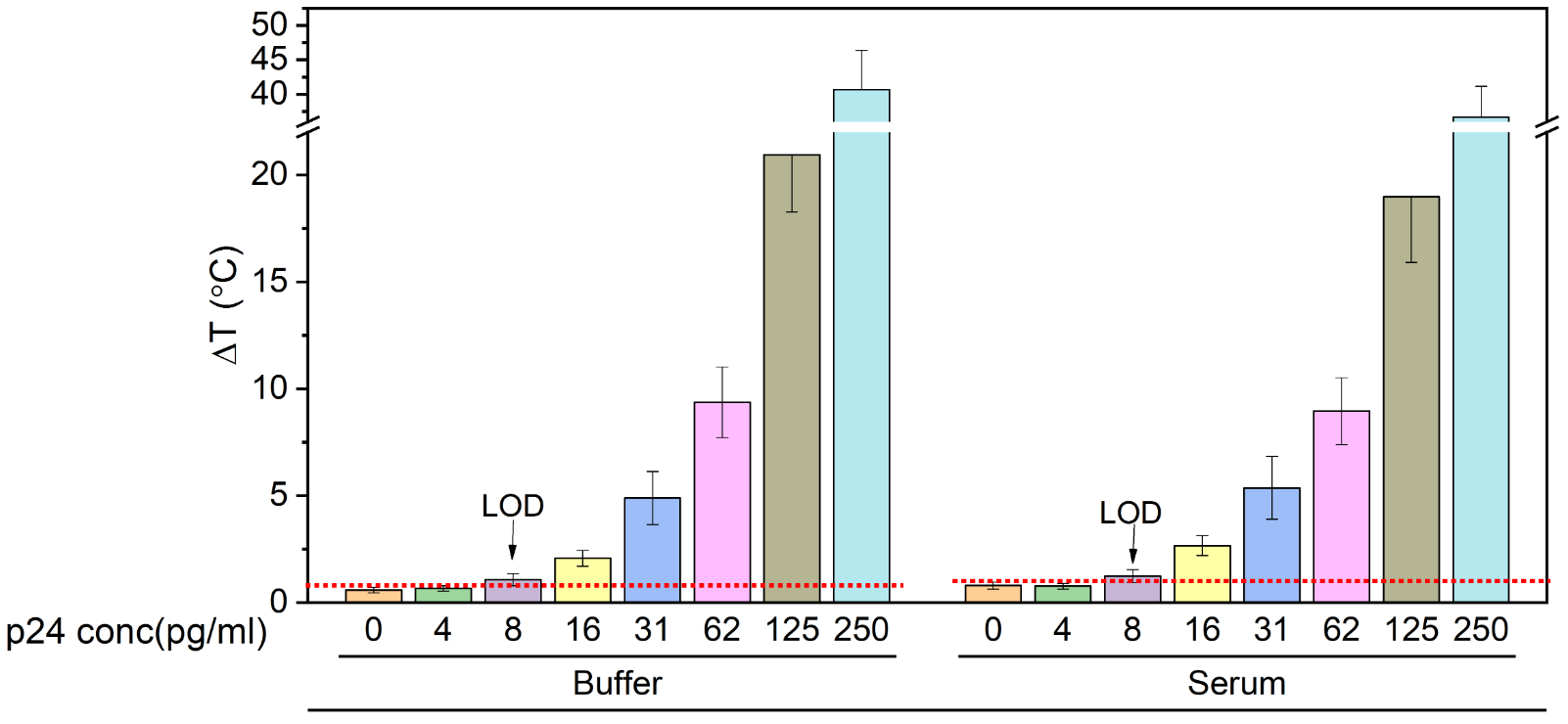


**Figure S2.** Testing of 2-fold serial dilution of p24 protein spiked in human serum with TCA LFAs using 100 nm gold spheres and 100 mW 532 nm laser. Red dotted line represents the cutoff thermal signal (average signal of blank samples plus three times standard deviation). The LODs were both 8 pg/ml for p24 protein spiked into buffer and serum.

**Reference**

1 Anfossi, L., Di Nardo, F., Giovannoli, C., Passini, C. & Baggiani, C. Increased sensitivity of lateral flow immunoassay for ochratoxin A through silver enhancement. *Analytical and bioanalytical chemistry* **405**, 9859-9867 (2013).

2 Rodríguez, M. O., Covián, L. B., García, A. C. & Blanco-López, M. C. Silver and gold enhancement methods for lateral flow immunoassays. *Talanta* **148**, 272-278 (2016).

3 Fu, X. *et al.* A SERS-based lateral flow assay biosensor for highly sensitive detection of HIV-1 DNA. *Biosensors and Bioelectronics* **78**, 530-537 (2016).

4 Hwang, J., Lee, S. & Choo, J. Application of a SERS-based lateral flow immunoassay strip for the rapid and sensitive detection of staphylococcal enterotoxin B. *Nanoscale* **8**, 11418-11425 (2016).

5 He, Y. *et al.* Ultrasensitive nucleic acid biosensor based on enzyme–gold nanoparticle dual label and lateral flow strip biosensor. *Biosensors and Bioelectronics* **26**, 2018-2024 (2011).

6 Lee, L. G., Nordman, E. S., Johnson, M. D. & Oldham, M. F. A low-cost, high-performance system for fluorescence lateral flow assays. *Biosensors* **3**, 360-373 (2013).

7 Loynachan, C. N. *et al.* Platinum nanocatalyst amplification: redefining the gold standard for lateral flow immunoassays with ultrabroad dynamic range. *ACS nano* **12**, 279-288 (2017).

8 Berlina, A. N., Taranova, N. A., Zherdev, A. V., Vengerov, Y. Y. & Dzantiev, B. B. Quantum dot-based lateral flow immunoassay for detection of chloramphenicol in milk. *Analytical and bioanalytical chemistry* **405**, 4997-5000 (2013).

9 Granade, T. C. *et al.* Rapid Detection and Differentiation of Antibodies to HIV-1 and HIV-2 Using Multivalent Antigens and Magnetic Immunochromatography Testing. *Clin. Vaccine Immunol.* **17**, 1034-1039, doi:10.1128/CVI.00029-10 (2010).
